# Electrical resistivity imaging (ERI) data for characterising crystalline basement structures in Abeokuta, southwestern Nigeria

**DOI:** 10.1016/j.dib.2018.07.034

**Published:** 2018-07-20

**Authors:** Ahzegbobor P. Aizebeokhai, Adenifesimi A. Oni, Kehinde D. Oyeyemi, Olubukola Ogungbade

**Affiliations:** Applied Geophysics Group, College of Science and Technology, Covenant University, Ota, Nigeria

## Abstract

This article consists of data sets for five (5) traverses of 2D electrical resistivity imaging (ERI) collected in Abeokuta, southwestern Nigeria. ABEM Terrameter (SAS1000/4000) system and dipole-dipole array were used for the data collection. RES2DINV computer program was used to invert the observed apparent resistivity data to obtain 2D inverse model images of resistivity distribution of the subsurface. The 2D resistivity images were used to characterise the subsurface and delineate the crystalline basement features of hydrological importance in the area.

**Specifications Table**

**Table:** 

Subject area	Geophysics
More specific subject area	Geoelectrical Resistivity
Type of data	Figure, DAT files and INV files
How data was acquired	Electrical Resistivity Imaging (ERI) using ABEM Terrameter (SAS1000/4000) system and dipole-dipole array
Data format	Raw, Inverted
Experimental factors	The observed apparent resistivity data sets were inverted to characterise the subsurface and delineate the basement features of hydrogeological importance.
Experimental features	Geophysical survey involving 2D Electrical Resistivity Imaging (ERI) was conducted.
Data source location	Abeokuta is between latitude $7{}^{\circ}60^{\prime }-7{}^{\circ}13^{\prime }N$ and longitude $3{}^{\circ}16^{\prime }-3{}^{\circ}25^{\prime }E$ in the crystalline basement complex, southwestern Nigeria.
Data accessibility	All the data sets are with this article.

**Value of the data**

• The 2D ERI dataset can be used for subsurface characterization and delineation of crystalline basement structures of interest in environmental, geotechnical and hydrogeological/hydrological investigations.
• The 2D ERI dataset can be used to characterise the weathering profile, and delineate the regolith thickness and fractured and weathered zones; these basement features are useful in groundwater exploration, foundation studies and geotechnical investigations in crystalline basement complex terrain.
• The data sets can be used to determine the spatial variability of basement aquifers, identify zones susceptible to weathering and fracturing; usually, groundwater preferentially accumulates in these zones and are therefore useful targets for borehole siting in basement terrain [e.g. [1], [2], [3], [4], [5]].
• The 2D ERI dataset can be integrated with other geophysical data sets such as induced polarization, magnetic, electromagnetic, ground penetrating radar, gravity and seismic data for detail subsurface characterization [e.g. [6], [7], [8]].
• The data set can be used for educational purposes, and for future research in hydrogeological, environmental and geotechnical studies. Similar data articles can be found be found in Refs. [9], [10], [11], [12].

## 1 Data

The attached files (Appendices A) consist of five traverses of 2D electrical resistivity imaging (ERI)) data for characterising and delineating crystalline basement structures. The raw datasets are presented in “dot DAT’ format (DAT files), while processed (inverted) datasets are presented in‘dot INV’ format.

## 2 Experimental design, materials and methods

### 2.1 Study area

**Fig. 1:**
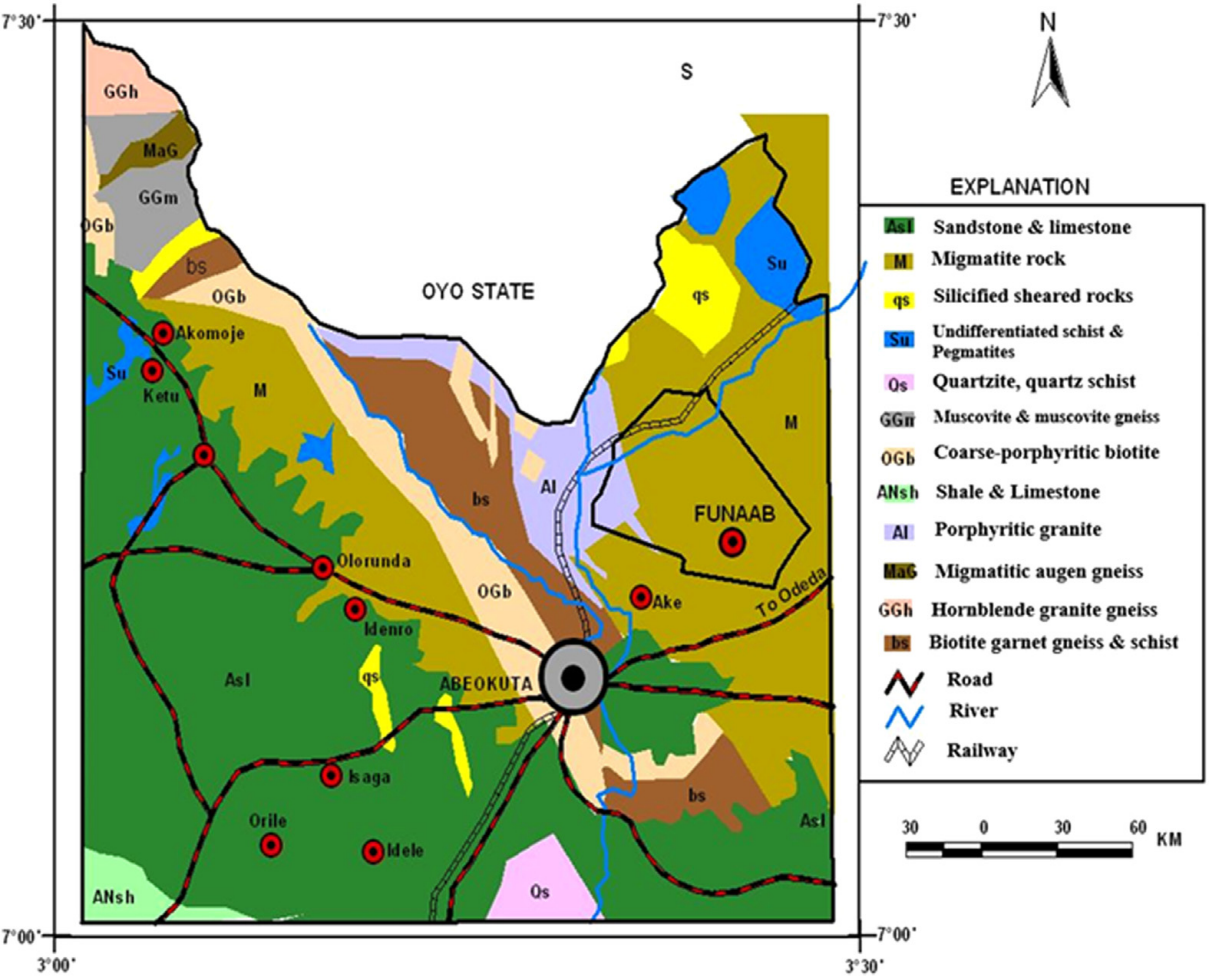
Location and geological of Abeokuta [1].

The study area lies between latitude $7{}^{\circ}60^{\prime }-7{}^{\circ}13^{\prime }N$ and longitude $3{}^{\circ}16^{\prime }-3{}^{\circ}25^{\prime }E$, and is located in Abeokuta, southwestern Nigeria. The topography is generally lowland characterised with sparsely distributed hills and knolls. The mean elevation is about 70 m above mean sea level. The climate is tropical humid marked by distinct dry and rainy seasons. Annual mean rainfall is greater than 2300 mm; monthly temperature ranges from 23 °C in July to 32 °C in February. Abeokuta is drained by Rivers Ogun and Oyan flowing along the strike of the basement rocks. Abeokuta is underlain by crystalline basement rocks of Precambrian age. The dominant rocks are biotite garnet gneiss, magmatic augen gneiss and migmatite which are essentially of migmatite-gneiss-granite complex. Fig. 1 shows the location and geological map of the study area.

### 2.2 Data acquisition

**Fig. 2:**
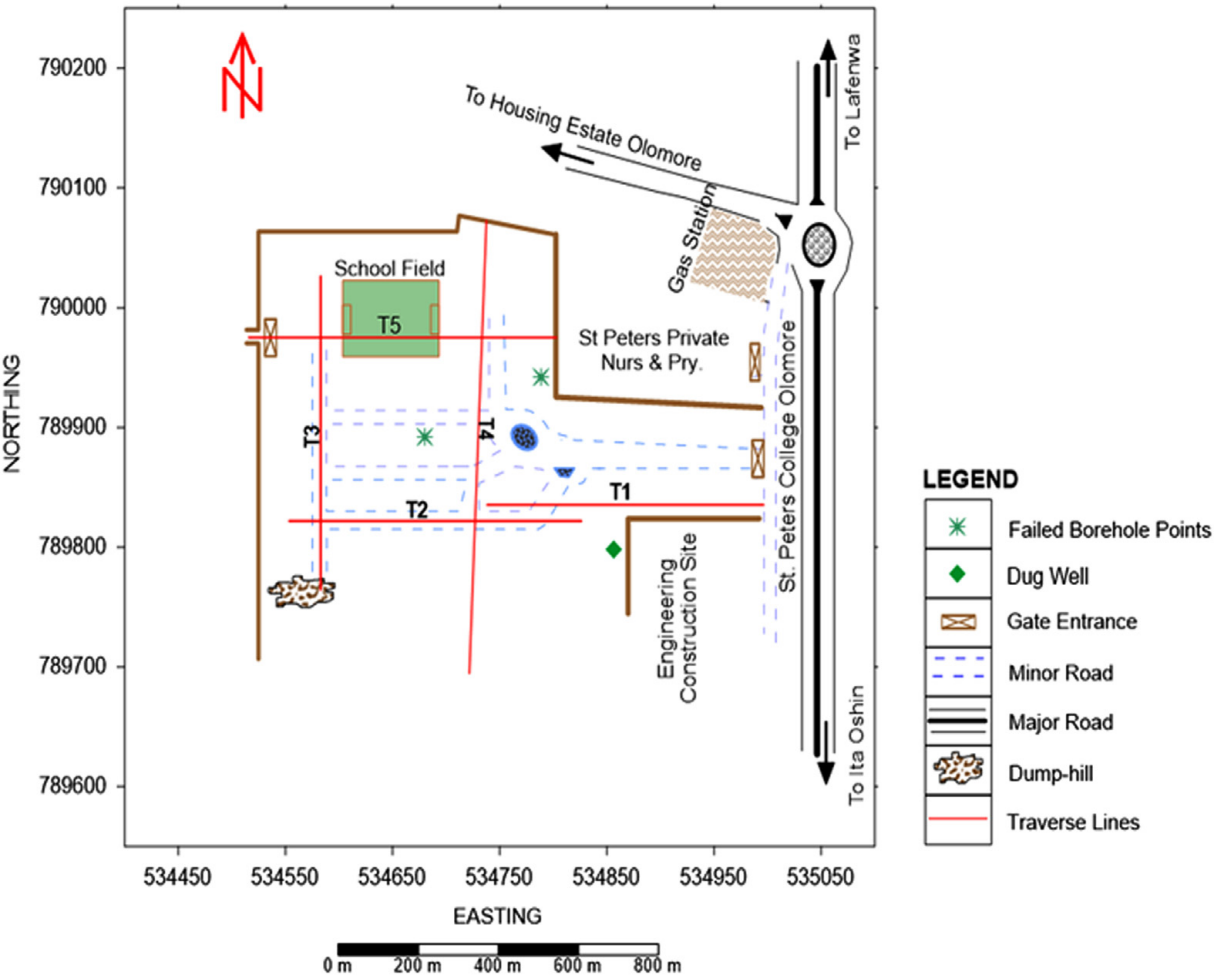
Base map of the study area indicating the 2D ERI traverses and borehole/well points.

The geophysical survey consists of 2D ERI; the datasets were measured using an ABEM Terrameter (SAS1000/4000) system during the month of February, 2018. The base map showing the locations of the 2D traverses is shown in Fig. 2. A total of five (5) 2D ERI were conducted using dipole-dipole array; the dipole-dipole array is sensitive to basement features such as faults, fractures and dykes which are commonly sought in hydrogeological investigations in basement terrain [13], [14], [15], [16], [17]. Dipole separation factor ranging from 1–4 was used for the 2D survey; minimum dipole spacing of 5 m was used for Traverses 1–3 and 5 while minimum dipole spacing 10 m was used for Traverse 4. Field techniques for geoelectrical resistivity survey have been discussed in several [e.g. [18], [19], [20], [21]].

### 2.3 Data processing

The observed apparent resistivity datasets were processed and inverted using RES2DINV inversion code [14], [20]. RES2DINV code uses a non-linear optimization technique to determine the distribution of 2D resistivity in the subsurface. Least-squares inversion technique with standard least-squares constraint (L_2_-norm), which minimizes the square of the difference between the observed and the computed apparent resistivity, was used for the inversion. The least-squares equation was solved using standard Gauss–Newton optimization technique; appropriate damping factors were selected based on the estimated noise level on the measured data.
